# Molecular Targeted Therapy and Immunotherapy for Myelodysplastic Syndrome

**DOI:** 10.3390/ijms221910232

**Published:** 2021-09-23

**Authors:** Paul Lee, Rita Yim, Yammy Yung, Hiu-Tung Chu, Pui-Kwan Yip, Harinder Gill

**Affiliations:** Division of Haematology, Medical Oncology and Haemopoietic Stem Cell Transplantation, Department of Medicine, LKS Faculty of Medicine, The University of Hong Kong, Hong Kong, China; pl85@hku.hk (P.L.); ritayim@hku.hk (R.Y.); u3558354@hku.hk (Y.Y.); u3557654@hku.hk (H.-T.C.); u3557642@hku.hk (P.-K.Y.)

**Keywords:** myelodysplastic syndromes, hypomethylating agents, treatment resistance, targeted therapy, immunotherapy

## Abstract

Myelodysplastic syndrome (MDS) is a heterogeneous, clonal hematological disorder characterized by ineffective hematopoiesis, cytopenia, morphologic dysplasia, and predisposition to acute myeloid leukemia (AML). Stem cell genomic instability, microenvironmental aberrations, and somatic mutations contribute to leukemic transformation. The hypomethylating agents (HMAs), azacitidine and decitabine are the standard of care for patients with higher-risk MDS. Although these agents induce responses in up to 40–60% of patients, primary or secondary drug resistance is relatively common. To improve the treatment outcome, combinational therapies comprising HMA with targeted therapy or immunotherapy are being evaluated and are under continuous development. This review provides a comprehensive update of the molecular pathogenesis and immune-dysregulations involved in MDS, mechanisms of resistance to HMA, and strategies to overcome HMA resistance.

## 1. Introduction

Myelodysplastic syndrome (MDS) is a hematological disorder characterized by ineffective hematopoiesis, cytopenia, and the propensity of clonal progression to acute myeloid leukemia. The Revised International Prognostic Scoring System (IPSS-R) is the most commonly used prognostic tool for predicting the risk of leukemic transformation and overall survival. Patients with lower-risk MDS (LR-MDS) are managed largely with supportive care and agents targeting improvements in anemia. Patients with higher-risk MDS (HR-MDS) are treated with the hypomethylating agents, azacitidine (AZA) or decitabine (DEC) [1]. Allogeneic hematopoietic stem cell transplant (HSCT) remains the only curative treatment for HR-MDS patients but is only limited to younger patients who are fit for allogeneic HSCT with suitable donors [2].

Clonal evolution studies of MDS stem cells have shown that CD123^+^ MDS malignant stem cells are more abundant in HR-MDS than LR-MDS patients and this stem population is enriched for protein synthesis activity despite they are quiescent [3]. The clinical heterogeneity of MDS is attributed to multiple genetic and epigenetic aberrations resulting in the dysplastic and proliferative features of MDS. In addition, MDS is characterized by a heterogenous immunopathology involving both innate and adaptive immunity. In general, cytopenia in LR-MDS is attributed to inflammation and apoptosis of progenitor cells and clonal expansion of dominant cell lineage in HR-MDS is associated with evasion of immune checkpoints [4].

## 2. Molecular Pathogenesis of MDS

Karyotypic abnormalities are seen in approximately 30–50% of patients with MDS and correlated with prognosis [5,6,7]. On the other hand, mutations are detectable by next generation sequencing (NGS) in more than 80% of patients with MDS with distinct mutation profiles observed in different MDS subtypes [8,9,10,11,12]. Multiple studies have tried to incorporate mutation profiling by NGS into the IPSS-R or WHO classification algorithm. Different demographic MDS datasets have been tested, and different studies have proposed the integration of different genes into their prognostic algorithms. In a European study done by Haferlach et al., 14 genes (*ASXL1*, *CBL*, *ETV6*, *EZH2*, *KRAS*, *LAMB4*, *NF1*, *NPM1*, *NRAS*, *PRPF8*, *RUNX1*, *STAG2*, *TET2* and *TP53*) were shortlisted to stratify MDS patients into four risk groups (low, intermediate, high, and very high risk) in combination with conventional scoring or as standalone scoring tools [13]. Similarly, Nazha et al. incorporated four genes (*EZH2*, *SF3B1,* and *TP53*) into their modified IPSS-R scoring system and achieved a significant improvement in the concordance index (C-index). Regardless of patient initial or subsequent therapies, the integration of mutation data also provides a dynamic platform for disease prediction [14]. For Asian MDS patients, Gu et al. proposed the mutation combined with the revised IPSS-R, namely MIPSS-R, taking into account the number of mutations and presence of *SF3B1* mutations. Upon risk level adjustment, 39% of patients achieved better clinical outcomes for receiving other treatment regimens following MIPSS-R in comparison with following the conventional IPSS-R [15]. More recently, MDS has been classified into eight distinct genomic subgroups based on their mutational and cytogenetic profiles, Group 0: MDS without specific genomic profiles; Group 1: MDS with *SF3B1* mutations and co-existing mutations (*ASXL1* and *RUNX1*); Group 2: MDS with *TP53* mutations and/or complex karyotype; Group 3: MDS with *SRSF2* and concomitant *TET2* mutations; Group 4: MDS with *U2AF1* mutations associated with deletion of chromosome 20q, and/or abnormalities of chromosome 7; Group 5: MDS with *SFSF2* mutations and co-existing mutations in other genes (*ASXL1*, *RUNX1*, *IDH2*, and *EZH2*); Group 6: MDS with isolated *SF3B1* mutations (or associated with mutations of clonal hematopoiesis and/or *JAK/STAT* pathways genes); and Group 7: MDS with AML-like mutation patterns (*DNMT3A*, *NPM1*, *FLT3*, *IDH1*, and *RUNX1* genes) [16]. These genomic groups correlated with the demographics, clinical, and hematological features, pathological classification and the overall survival [16].

**Figure 1:**
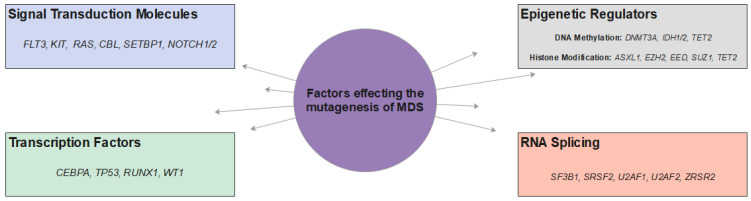
Molecular pathogenesis of MDS. Signal transduction molecules refer to gene mutations resulting in alteration to proliferative or apoptotic effects. Transcription factors and epigenetic regulators exert effects at both transcriptional and translational levels due to aberrations in RNA splicing, DNA methylation, and histone modification.

The functional impact of mutations affecting signal transduction, transcriptional regulation, epigenetic regulation and RNA splicing molecules (Figure 1) will be discussed in details [17,18].

### 2.1. Signaling Molecules and Pathways

#### 2.1.1. FLT3

The fms-like-tyrosine kinase III (FLT3) is a transmembrane tyrosine kinase, highly expressed on hematopoietic progenitor cell surfaces and is crucial for the development of hematopoietic stem cells (HSC) and progenitor cells [19]. This signaling pathway, stimulated by FLT3, regulates cellular processes such as cell division, survival, and growth in hematopoietic progenitor cells. *FLT3* mutations reported in MDS are mostly internal tandem repeat (*FLT3*-ITD) located on exon 14 or 15 of chromosome 13q12 encoding the juxtamembrane domain, or, less commonly, a single point mutation of the tyrosine kinase domain (TKD) [20]. *FLT3* mutations are well documented in AML with a prevalence of 30% but *FLT3* mutations occur at a much lower rate (~6%) in MDS [20,21,22]. Patients who carry *FLT3* mutations are usually presented with a more adverse clinical course with a high risk of leukemic transformation [23]. *FLT3* mutation is more common in younger patients and MDS with excess blast-1 or excess blast-2 (MDS-EB-1 or MDS-EB-2) [24]. *FLT3* mutations lead to constitutive activation of *FLT3* and downstream STAT5 signaling pathways. Exome sequencing done by Kim et al. revealed that the acquisition of *FLT3* mutations in HSCs or hematopoietic progenitor cells (HPC) can drive the formation of leukemic stem cells (LSC) [25]. It was proposed that mutation acquisition can originate from the immature hematopoietic compartment, resulting in the accumulation of non-self-renewing blasts, which inevitably promote the transformation into AML [26,27].

#### 2.1.2. KIT

*c-KIT* proto-oncogene encodes a type III tyrosine kinase (*KIT*) is also known as CD117, and it is a mast/stem cell growth factor receptor (SCFR) influencing HSC survival, proliferation, and differentiation into the hematopoietic lineages [9,28]. There are three major protein domains involved in activities of KIT and downstream activation of PI3Kinase, Ras, and MAPK signaling. They are the extracellular membrane domain (EM) at exon 8, the juxta-membrane domain at exon 11, and the tyrosine kinase domain (activation loop) at exon 17. Gain-of-function mutations of these domains have been reported in other malignancies and some are also found in MDS patients [28,29]. *KIT* mutations are predominantly observed in patients with MDS-EB-1, MDS-EB-2, and secondary AML [29]. Among these mutations, most are gain-of-function mutations leading to ligand-independent activation of the KIT signaling, such as the D816V or D816Y of the tyrosine kinase domain.

These pathogenic mutations are likely associated with upregulation of KIT expression at the transcript and protein levels, observed in MDS patients, and the overexpression is more noticeable in patients with MDS-EB-1, MDS-EB-2, and secondary AML. In vitro studies have further shown that *KIT* expression can be induced by interleukin-3 (IL-3) and erythropoietin (EPO), with or without stem cell factor (SCF) expression on cell isolated from MDS patients [30]. In fact, depletion of KIT with humanized monoclonal antibody ablates the MDS HSCs in both LR- and HR-MDS xenograft models. Yet this also permits the subsequent engraftment of healthy HSCs, which is evident by robust engraftment of human B-cells and T-cells post-depletion of KIT. This confirms the stem cell’s regulative property of KIT in MDS and AML, and hence the oncogenic property of *KIT* mutations [31].

#### 2.1.3. RAS

RAS proteins are encoded by three proto-oncogenes (*H-RAS*, *K-RAS*, and *N-RAS*) that regulate differentiation and growth of many cell types, including myeloid cells. *RAS* proteins are membrane-associated GTPases that regulate serine or threonine kinases of the MAP kinase cascade and mutations of *RAS* often constitutively activate RAS/MEK and RAS/PI3K signaling by accumulating intercellular RAS GTP [32]. Large scale studies have consistently reported RAS signaling deregulation due to mutations of *RAS* genes in MDS. *RAS* mutations are detected at an overall rate of 3–5% with *N-RAS* genes being the most frequently mutated and *K-RAS* accounting for the remaining RAS mutations [13,32]. *N-RAS* mutations at codon 12 account for 55% of all *RAS* mutations followed by *N-RAS* mutations at codons 31 and 61 (12% each) and *K-RAS* mutation at codon 12 (21%) [33]. Patients with *RAS* mutations also show higher white cell count and bone marrow blast percentage. Although the frequency of *RAS* mutations in MDS is much lower than that in CMML or AML, mutations of *RAS* in MDS patients, in particular *N-RAS*, have been found associated with shorter survival and higher risk of transformation into AML [33,34,35].

#### 2.1.4. CBL

*CBL* plays an important role in tyrosine kinase signaling. CBL can activate signaling complexes and positively regulates downstream signal transduction components [36]. However, *CBL* can also negatively regulate tyrosine phosphorylation through controlling the ring domain and tyrosine-kinase-binding (TKB) domain to induce ubiquitination. CBL also promotes E3 ligase catalytic activity on lysine residue in substrate proteins. As a result, lysine ubiquitination triggers proteasomal degradation and recycling in endosomes [37]. CBL controls protein degradation of several important proteins in myeloid neoplasms, including c-Kit, FLT3, and STAT5, which STAT5 is a key downstream component of the *JAK2* signaling. Not surprisingly, *CBL* mutations have been reported in 10% of patients with MDS/myeloproliferative neoplasm (MPN) overlap syndrome and these patients display clinical features such as splenomegaly, monocytosis, and anemia [38,39]. In MDS/MPN overlap syndrome, Sanada et al. also discovered a *CBL* gain-of-function mutation can contribute to loss of the ubiquitin E3 ligase activity, thereby prohibiting ubiquitin-mediated degradation of tyrosine kinases, resulting in constitutive activation of tyrosine kinase signaling. Moreover, they have also reported *CBL* mutations as acquired uniparental disomy (aUPD) in 31.5% of the patients studied, and it is more common in MDS/MPN than MDS patients, where both copies of a chromosome pair or parts of chromosomes originated from a single parent [36]. Conventionally, aUPD is a mechanism by which pathogenetic mutations in cancer may be reduced to homozygosity. This process is typically described in loss-of-function mutations but is rarely reported in gain-of-function mutations. Therefore, this postulation provides novel insight on how the gain of oncoprotein resembles the loss of tumor suppressive gene [37].

#### 2.1.5. SETBP1

*SETBP1* gene is localized to chromosome 18q21.1 which encodes SET binding protein 1 and binds with SET nuclear oncoprotein to form a heterodimer and this complex suppresses SET from proteasomal degradation and downstream activity of tumor suppressor protein phosphatase type 2A (PP2A) [40,41]. Makishima et al. showed that *SETBP1* mutation can affect tumor formation [40,42,43]. In MDS, multiple studies have reported *SETBP1* mutations and the hotpot mutations localizes to codon 858–871. Changes like D868N, E858K and G870S belong to the SKI homologous region are predicted to be responsible for altering binding to SET [44]. While the mutation frequency varies from 2% to as high as 50% and is more commonly observed in LR-MDS, meta-analysis has shown that patients with *SETBP1* mutation have significantly shorter survival. Thol et al. further reported a significantly higher rate of relapse within one year, and Shou et al. further showed that *SETBP1* mutation is associated with poor prognosis in patients with MDS and CMML [8,44,45,46,47,48,49]. These mutations are somatic gain-of-function mutations associated with -7/del(7q) and are also enriched in patients with *ASXL1* mutations as concurrent mutation, conferring a higher risk of transformation into AML [48]. In terms of function, these mutations, especially D868N and G870S, are proven to be pathogenic, as they suppress self-ubiquitination, hence inhibiting self-degradation and eventually leading to impaired apoptosis and differentiation.

In vitro studies have shown the increased clonal expansion can be further enhanced with additional *ASXL1* mutation, and *ASXL1* mutation shows a significantly higher rate of co-mutation with *SETBP1* in patients [48,50]. This increases expression of mutant SETBP1 oncoprotein that favors the transformation of MDS cells into AML. One of the downstream targets of these mutants involved in MDS pathogenesis is the repression of TGF-β signaling. Several lysine residues on histone H3 and H4 around the promoter regions of multiple TGF-β pathway genes undergo aberrantly reduced acetylation. The dysregulated TGF-β signaling in double mutant mice also shows higher engraftment, dysfunctional differentiation and uncontrolled cell cycle. However, these abnormalities are reversible using the HDAC inhibitor Vorinostat [51].

#### 2.1.6. NOTCH 1/2

*NOTCH1/2* is a highly conserved cell surface signal transducer that regulates developmental and cell fate. In particular, NOTCH receptors are expressed by early HSC whereas NOTCH ligands are expressed by bone marrow stromal cells. Collectively, this forms a microenvironment providing the signal required for progenitor differentiation and proliferation [52]. The involvement of NOTCH dysregulation in MDS involves the dysplastic microenvironment with a suppressed NOTCH signaling activity followed by downregulation of delta-like-1 (DLK-1), which is an early adipogenic cell fate inhibitor. This causes an impaired colony-forming unit fibroblast in vitro, and this potentially contributes to a biased adipogenic differentiation of bone marrow stromal cells, also observed in bone marrow isolated from MDS patients [53].

In addition to impaired bone marrow microenvironment, NOTCH1-mediated suppression also contribute to HSC inadequate differentiation into progenitor subpopulations MPP3 (myeloid) and MPP4 (lymphoid), leading to dysplasia and selective clonal expansion in a murine model. During leukemic transformation or under cellular stress, *NOTCH1*-mediated signaling is suppressed and the differentiation capacity of lymphoid MPP4 into mature lymphocytes is suppressed. MPP4 also diverges and contributes to myeloid cell expansion alongside with activated myeloid MPP3, leading to the accumulation of myeloid blast cells [54]. Therefore, it is not surprising that the loss-of-function mutation of NOTCH signaling genes could contribute to MDS and AML transformation.

In fact, *NOTCH1/2* mutations have been reported at a frequency of <5% in MDS and 12% in AML patients, but the direct evidence of *NOTCH*1/2 mutation implicated in MDS is limited [8,55,56]. Despite there being no significant correlation of *NOTCH1/2* mutation with IPSS-R, *NOTCH1* mutation is preferentially observed in HR-MDS [8]. With the postulation that MDS cells are subjected to apoptosis hence cytopenia is at least partially attributed to NOTCH signaling, Fu et al. performed in vitro studies by stimulating primary MDS cells with recombinant NOTCH ligand, hypothesizing that NOTCH signaling activation would suppress apoptosis. However, their results indicated that NOTCH ligands do not improve cytopenia features. The forced activation of NOTCH in vitro further decreases the formation of neutrophil colonies and increases the proportion of granulocyte-macrophage (GM) colonies and macrophage (M) colonies, proposing that NOTCH signaling is dysregulated in dysplasia during MDS pathogenesis [57].

#### 2.1.7. Other Signal Transducers

*CSF3R* is a transmembrane receptor for colony stimulating factor 3 (CSF3), mediating normal growth in myeloid progenitors. Under normal conditions, G-CSF binds to CSF3R for promoting growth and survival of myeloid precursor cells to neutrophils. *CSF3R* mutation is rarely seen in MDS. *CSF3R* mutations fall into two types: (1) nonsense or frameshift mutations resulting in premature truncation of cytoplasmic receptor and (2) point mutations at the extracellular domain of *CSF3R* [58]. It is shown that CSF3R is sensitive to JAK2 inhibitors, such as ruxolitinib [59]. Alternatively, truncated *CSF3R* truncated mutations are sensitive to SRC kinase inhibitors, such as dasatinib. Nevertheless, whether *JAK2* or *SRC* inhibitors are of benefit to patients with *CSF3R* mutations remains to be investigated, since the majority of studies conducted regarding CSF3R are related to CML. The true incidence of this mutation appears to be much less in MDS, but it still presents as a significant mutation for the disease benchmark [60].

Janus kinase 2 (*JAK2*) is a non-receptor tyrosine kinase that regulates signaling pathways through *MPL*, *TpoR, EpoR,* etc. *JAK2* is crucial for normal hematopoiesis and *JAK2V617F* mutation is found in 2–5% of MDS cases. An increased incidence of thrombosis, hemorrhage, and fibrotic transformations are associated with the presence of the mutant allele in MDS. However, *JAK2V617F* mutation is less implicated in MDS in comparison with other myeloid malignancies, especially MPN, where *JAK2V617F* mutation is considered to be the driver mutation of MPN [61,62,63].

### 2.2. Transcription Factors

#### 2.2.1. TP53

*TP53* is a tumor suppressor gene located at chromosome 17p13.1, containing 11 exons, and is a transcription factor responsible for cell regulation and induction of apoptosis upon exposure to UV. *TP53* is one of the most studied genes in cancers [64,65]. Somatic *TP53* mutations are more frequently seen in HR-MDS, therapy related MDS, and MDS transformed leukemia. Complex karyotypes with deletions of chromosome 5, 7, and 17 (i.e., *TP53*) show a strong correlation with poor risk IPSS with morphological features, such as increased blast cell percentage and thrombocytopenia [66]. Deletion of chromosome 5q results in the loss of a “commonly deleted region” (CDR), which comprises 41 genes, including *RPS14*, and haploinsufficiency of *RPS14* is associated with erythroid failure in MDS [67]. Another gene that is closely regulated with *TP53* is the Murine Double Minute-2 (*MDM2*). Under normal conditions, MDM2 is free to bind p53 and the binding of MDM2 with p53 triggers p53 ubiquitination and subsequent degradation.

Cytotoxic stress can activate the phosphorylation of both MDM2 and p53 via the ATM-Chk1 or ATM-Chk2 axes. This promotes a range of post-translational modifications of MDM2, including acetylation, methylation, and SUMOylation, which in turn contribute to p53 accumulation. The abnormal accumulation of p53 leads to cell cycle arrest, impaired DNA repairs, senescence, and apoptosis [68,69]. *TP53* defects adversely influence MDS clinical outcome and the treatment response rate, such as resistances to HMA. Therefore, new therapeutic approaches, such as immune checkpoint inhibitions, are being developed for these patients, which will be reviewed in the later sections (Section 4) of this review [64].

Clonal evolution analysis of *TP53* mutation in MDS has been described by da Silva Coelho et al. and both linear and branching patterns of evolutions were detected in the study [70]. In particular, *TP53* mutation was found to be a branching clone as de novo mutation during treatment with lenalidomide, and TP53 is responsible for the drug resistance in these MDS patients [70]. Similarly, clonal evolution study done by Makishima et al. also proposed *TP53* mutant clone as a namely “type 2 mutations”. This group of clonal mutations are enriched in HR-MDS patients and is predicted with limited impact on AML transformation in comparison with “type 1 mutations”, including *FLT3*, *RAS,* and *IDH1/2,* which harbor a significant impact on patient survival and AML progression. However, *TP53* is also reported as a unique mutation, being mutually exclusive with other mutations, suggesting its potential role as a driver of mutation in MDS [71].

#### 2.2.2. RUNX1

*RUNX1* is a transcription factor that regulates HSC maturation into mature blood cells. *RUNX1* is located on chromosome 21q22.12 and encodes an alpha subunit of the core-binding factor (CBE) complex [72]. This regulates transcription activities for key genes in differentiation, growth, and survival pathways. *RUNX1* is commonly reported with somatic mutations and a majority of *RUNX1* mutations found in MDS are small SNV or INDEL [73]. The mutation frequency of *RUNX1* in MDS is 10% and patients with *RUNX1* mutations have higher neutrophil counts, a higher frequency of -7/7q deletion, and shorter overall survival [74].

This is concordant with the clonal sweeping model proposed by Makishima et al. regarding the disease progression of MDS from LR-MDS to HR-MDS, with *RUNX1* mutation being one of the “type 2 mutations” [71]. Furthermore, *RUNX1* mutant clones can be gradually replaced by the acquisition of another group of mutations, namely “type 1 mutations”, as patients develop AML during the process of clonal evolution, in which “type 1 mutations” are associated with poorer survival and a faster rate of AML transformation [71]. In this study, two models of clonal evolution can be responsible for the mutation dynamics in different patients. They are clonal sweeping or linear evolution, of which linear evolution is the most common model and is characterized by the stepwise arising of a small subclone under the presence of a pre-existing dominant mutant clone and further expands during disease progression [71]. On the other hand, clonal sweeping refers to the pre-existence of multiple mutation clones, but one clone with the highest fitness (e.g., driver mutations) is selectively swept and dominates over other passenger mutations with less or no effects inside the tumor [75]. Regarding large genetic lesions involving *ASXL1* mutation in MDS, some studies have proposed -7/7q deletion as a secondary event to *ASXL1* mutation, which can cause activation of the RTK-RAS pathway, promoting leukemic transformation [74,76].

#### 2.2.3. WT1

Wilms tumor 1 (*WT1*) is a tumor-suppressor gene coding for a zinc finger transcription factor located on chromosome 11p13, and was originally identified in Wilms’ tumor [77,78]. *WT1* mutations have been reported in 3–4% of MDS cases and sequencing studies have also shown that the presence of *WT1* mutations favors MDS transformation into AML [79]. *WT1* shows exceptionally low to absent expression in normal tissues while it is highly expressed in a wide range of malignant neoplasms with its key role being in regulating tumor cell proliferation. Around 60% of patients with MDS with single lineage dysplasia (MDS-SLD) overexpress *WT1* in both bone marrow and peripheral blood samples, and *WT1* expression level shows a good correlation with WHO clinical classifications and IPSS scores [80,81]. An in vivo study characterizing the hematologic phenotype of *WT1* mutation demonstrated that, in the presence of the *WT1* mutation, hematopoietic progenitor cells in mice significantly expand with an aggressive MDS/MPN phenotype, manifesting into anemia and erythroid dysplasia with a decreased survival [81].

#### 2.2.4. CEBPA

*CEBPA,* which encodes C/EBPα, is a CCAAT enhancer-binding protein that plays an important role in myelopoiesis, especially the differentiation from common myeloid progenitors (CMPs) into granulocyte-monocyte progenitors (GMPs) and eventually into mature granulocytes and monocytes. It possesses 14 enhancer regions and distinct enhancer combinations are active in different *CEBPA*-expressing tissues. Conformational studies of 3D genomic have shown that *CEBPA* localizes to a 170-kb conserved topological-associated domain (TAD) on chromosome 19 and two enhancers located +21kb region located 3′ of the *CEBPA* are myeloid specific with the highest promoter interaction in *CEBPA*^+^ myeloid cell lines in comparison with *CEBPA^−^* lymphoid cell lines. This myeloid-specific chromatin conformation is also exclusively marked with active histone H3K27ac in differentiated neutrophils and monocytes. Moreover, the enhancers are occupied by the HSC specific transcription factors, including *GATA*, *RUNX1*, and *PU1*, evident by positive detection of these motifs on this specific region. Deleting one of the two enhancers in vivo thereby indirectly suppresses myeloid specific *CEBPA* and has a severe impact [82]. This causes demolished myelopoiesis and hence severe neutropenia and insensitivity to stimulation by G-CSF and GM-CSF, illustrating the functional importance of *CEBPA* in neutropenia, and hence infections in MDS [82,83]. In addition to distant enhancer dysregulation that causes suppressed *CEBPA* activity, in-frame but loss-of-function mutations of the *CEBPA* gene body itself are reported in MDS or MDS transformed AML. At a much lower rate than de novo AML (~5.4% vs. 17.2%), and unlike AML which can have double mutations at both N- and C-terminus of C/EBPα, MDS patients only have one of the mutations [84,85]. Mutations at N-terminus suppress the activation capacity of C/EBPα by its direct binding to the target promoter or by its heterodimerization with C/EBPα. On the other hand, mutations at the C-terminus disrupt the basic zipper structure and part of the DNA binding domain, likely suppressing C/EBPα activity indirectly through disrupted interaction with other transcription factors, such as *PU1* [84].

#### 2.2.5. Other Transcription Factors

*NPM1* (Nucleophosmin 1) is located on chromosome 5 (q35.1) and is associated with nucleolar ribonucleoprotein, which is responsible for the biogenesis of ribosomes, mRNA processing, and chromatin remodeling [86,87]. Mutations of *NPM1* are detected in 20–30% of AML cases, but are less frequent in MDS (9%) [88,89]. *NPM1* mutations are rare in MDS/MPN [90]. Bains et al. documented *NPM1* mutations from a large cohort study in which the results were strongly associated with normal karyotypes and HR-MDS [88]. Due to the rarity of *NPM1* mutation in MDS, there are no robust molecular or clinical data to further understand disease evolution in *NPM1*-mutated MDS.

### 2.3. RNA Splicing

**Figure 2:**
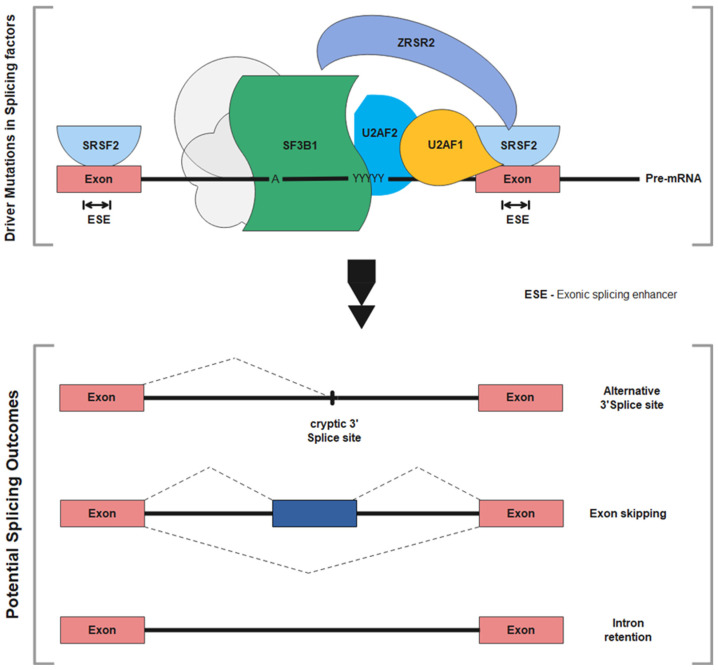
Driver mutations in splicing factors. The diagram shows key driver mutations in splicing factors in myelodysplastic syndrome and their potential splicing outcomes if one or more of these splicing factors are mutated.

In studies applying exome sequencing and targeted sequencing in MDS, it has been revealed that multiple RNA splicing mutations are implicated in its pathogenesis [91]. In normal cells, RNA splicing machinery begins with the pre-messenger RNA (pre-mRNA) intron removal and fusion of exons to form a mature mRNA. Spliceosomes are used to fuse 5′-mRNA splice site upstream exon to 3′-mRNA splice sites. They consist of the assembly of five small nuclear ribonucleoproteins (snRNPs) via their sequential binding to the pre-mRNA; hence, leading to the initiation of RNA splicing. This is achieved through the recognition of the 5′-mRNA splice site by U1 snRNP while the 3′ site is recognized by the U2-auxiliary factor (U2AF) [92,93]. The U2AF protein consists of a U2AF35 (U2AF1) subunit and a U2AF65 (U2AF2) subunit. U2AF protein binds to SF3B1 through a splicing factor (SF1) to form a heterodimer complex [94]. The U2AF35/U2AF65 heterodimer has a high affinity for ZRSR2 (Zinc finger RNA binding motif and serine/arginine rich 2) and SRSF2 (serine/arginine rich splicing factor), which binds to the polypyrimidine tract located at the 3′ splice site. SRSF2 involves the removal of introns from the primary transcript and is responsible for influencing patterns for alternate splicing. Any alterations to the spliceosome complex can result in a change in splicing specificity, leading to alternative splicing outcomes (Figure 2) [95,96].

In MDS, concurrent spliceosome gene mutations, such as *SRSF2* and *SF3B1,* are associated with HR-MDS, higher marrow blast percentage, and dysregulations of RNA splicing and DNA methylation pathways [13]. Splicing factor mutations at 3′ mRNA splice sites are common in MDS and around 60% of MDS cases harbor mutations related to splicing factors. Frequently mutated splicing genes amongst MDS patients include *SRSF2* (~12.4%). Another 20% of MDS patients carry multiple mutations, such as *U2AF35*, *U2AF65*, *SF3B1,* and *ZRSF2* [97,98]. In particular, functional studies of *U2AF35* mutation with other splicing factors have shown that splicing impairment, including intron retentions, induces mRNA splicing pathways and eventually growth impairment [99]. The *SRSF2* mutation clusters around hotspot residue Pro95 are associated with epigenetic *TET2* mutations [97]. *SF3B1* mutations have been reported as a potential initiating event in defining sideroblastic anemia [11,100]. Computational analysis shows recurrent driver mutations, such as *SF3B1,* are associated with 5q deletion (del(5q)) in patients with MDS-RARS [100]. *SF3B1* mutations are found in 68% and 81% in patients with MDS with ring sideroblasts and MDS/MPN with ring sideroblasts and thrombocytosis (MDS/MPN-RS-T), respectively. In vitro and in vivo experiments have also demonstrated the association of *SF3B1* mutations with altered iron distribution in MDS patients ring sideroblasts and haploinsufficiency of *SF3B1* is sufficient to cause ring sideroblasts transformation [98]. Woll et al. identified a high mean variant allele frequency (VAF) of *SF3B1* (30.8%), showing that the *SF3B1* mutation is present in dominant MDS clones. Hence, this suggests the mutation itself originates from cells actively propagating in the MDS clones [101]. Moreover, *SF3B1* mutation in MDS patients is often associated with alternative splicing of *SLC25A37*, a crucial importer for iron in mitochondria [102]. Based on single cell clonogenic data, it has been proposed that a sequential acquisition of genetic lesions with *SF3B1* in one of the mutated major clones at the early stages of MDS is implicated for subsequent AML transformation [103].

Mian et al. demonstrated that *SF3B1* mutations in MDS with ring sideroblast can arise from HSCs during subclonal evolutions during disease pathogenesis [103]. Clonal analysis reveals the MDS mutational architecture displays an overall dominance of the *SF3B1* mutation in primary CD34^+^ bone marrow, hemogenic endothelial cells (HEC), and Long Term culture initiating cells (LTC) [103]. For the functional impact of *SRSF2* mutation in MDS, Kim et al. reported that *SRSF2* mutation is associated with alternative splicing of epigenetic regulator *EZH2*. This induces a nonsense-mediated decay of *EZH2* transcript, eventually impairing HSC differentiation. In vitro experiments rescuing intact *EZH2,* on the other hand, can restore the hematopoietic defects induced by mutant *SRSF2*, suggesting the crosstalk of compound mutations of different groups of genes in MDS pathogenesis [104]. Moreover, patients with splicing factor mutations alone have been reported to have better overall survival than those with additional mutations, such as cell signaling/transcriptional regulator, epigenetic modifiers, or other members of the splicing machinery [105].

### 2.4. Epigenetic Dysregulation-DNA Methylation

**Figure 3:**
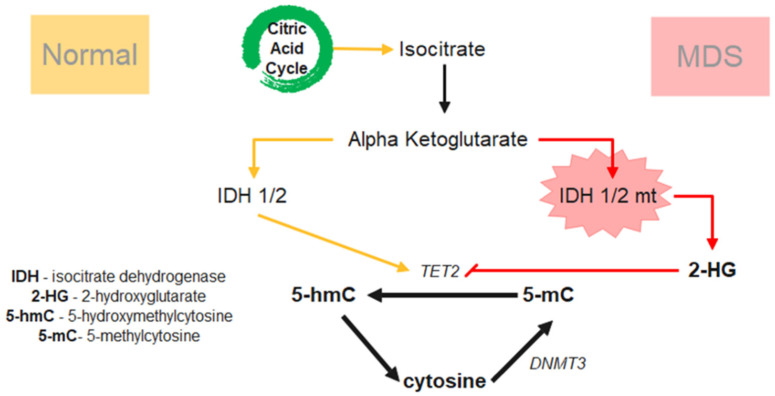
Mechanisms of DNA methylation under epigenetic dysregulation in MDS. Under normal circumstances, IDH1/2 promotes the *TET2* genes to perform the conversion of 5-mC to 5-hMC, triggering DNA methylation. However, *IDH1/2* mutations are often seen in MDS causing the inhibition of *TET2*, resulting in global hypomethylation but loci-specific hypermethylation.

Demethylation of the cancer genome is the principal rationale of using HMA in myeloid malignancies. CpG methylation within gene promoters is a major epigenetic transcriptional silencing mechanism that is frequently dysregulated. In particular, transcriptional inactivation involving DNA methylation is primarily attributed to the methylation of CpG dinucleotides at the promoter region and gene bodies. This regulatory machinery is strictly regulated by DNA-methyltransferases (DNMTs), ten eleven translocations (TET) enzymes, and isocitrate dehydrogenases (IDHs) [106,107]. *DNMT3A* enzymatically adds a methyl group to 5′ cytosine at the CpG dinucleotide resulting in DNA methylation while the removal of the methyl group can be mediated by TET family proteins during demethylation [108]. TET2 plays a pivotal role in the oxidation of 5-methylcytosine (5mC) to 5-hydroxymethylcytosine (5hmC). In the citric acid cycle, IDH1/2 normally catalyzes isocitrate to α-ketoglutarate (α-kg) while TET2 relies on α-kg to function normally. When *IDH* mutation occurs, it gains an additional function to produce 2-hydroxyglutrate (2-HG) which inhibits the TET family proteins directly. *IDHs* mutations (e.g., at arginine 132 amino acid) are associated with elevated serum levels of 2-HG enantiomers (D-2-HG) in MDS patients, resulting in a reduction of 5hmC levels, and eventually hypermethylation (Figure 3) [109,110]. *IDH1/2* mutations are present in approximately 5–12% of MDS patients with *IDH2* mutations occurring at a higher frequency than *IDH1* [111]. Despite *IDH1/2* has a lower incidence in MDS compared to AML, its occurrence increases over time with disease progression [111]. Other studies have shown early driver mutations in epigenetic modifiers can have co-mutation with spliceosomes genes, e.g., mutation of *IDH2, EZH2* can couple with mutations of *SF3B1, U2AF1, RUNX1,* and *STAG2*, to dictate disease evolution with distinct clinical phenotypes in MDS [62,112].

In MDS, mutations of these epigenetic modifiers and spliceosomes are found to be associated with inflammasome signaling activation. This is evidenced by the finding that epigenetic dysregulation is associated with activation of NOD-like receptor protein 3 (NLRP3) inflammasomes, followed by increasing levels of damage-associated molecular patterns (DAMPs) in MDS. DAMPs are high mobility group proteins, such as B1 and alarmin S100 proteins, which are responsible for sensing the presence of Toll-like receptors (TLRs). Increased levels of DAMPs and NLRP3 inflammasomes activation are enriched in LR-MDS patients [113,114,115,116]. MDS stem cells are specifically susceptible to DAMPs because they overexpress TLRs, as well as signal transducers, such as *IRAK1* and *TRAF6* [117,118]. These mutations increase the production of pro-inflammatory cytokines, such as IL-6 and Type 1 IFN-α. The dysregulated inflammasome with S100A9 and NLRP3 having a role to play activates β-catenin signaling, eventually resulting in pyroptosis [113,119,120]. Ligation of S100A9 with TLR4 induces NF-κB-mediated transcription of pro-inflammatory cytokines, including pro-interleukins IL-1β, IL-18, and other inflammasome components [112]. The active NLRP3 inflammasome directs caspase-1-dependent conversion of pro-IL-1β/IL-18 into active forms resulting in pyroptosis [112,113].

### 2.5. Epigenetic Dysregulation—Histone Modification

#### 2.5.1. EZH2, EED and SUZ12

**Figure 4:**
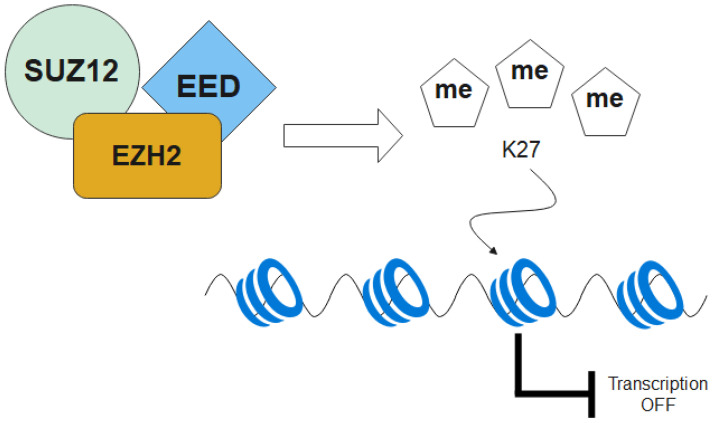
Mechanism of histone modification in MDS. Under normal circumstances, the PRC2 complex promotes methylation by regulating H3K27me resulting in transcriptional activation. However, loss-of-function mutation of *EZH2* causes PCR2 to malfunction resulting in HSC disorders.

Alterations in epigenetic processes, including DNA methylation and histone modifications, are well-known pathological events in MDS that self-renewal of HSC compartment is often dysregulated. Enhancer of the Zeste Homolog 2 (EZH2) is a protein subunit of the poly-comb repressive complex 2 (PRC2). PRC2 catalyzes methylation of histone H3 lysine 27 methyltransferases (H3K27me), hence maintaining transcriptional repression of genes involved with cell fate decisions. In MDS, *EZH2* mutations can result in a malformed PCR2 complex hence loss-of-function of H3K27me and impede normal function of the other two subunits *EED* and *SUZ1* (Figure 4). While mutation of EED and SUZ12 in MDS are rare (<1%), *EZH2* mutation is common and is associated with poor prognosis in MDS. In addition to male predominance of *EZH2* mutation in MDS, both *EZH2* mutation and loss of EZH2 protein expression independently correlate with inferior survival and R-IPSS score [121,122]. Despite the fact that survival is not affected in vivo, murine models with *EZH2* double knockout develops myelodysplastic phenotypes evident by morphologic dysplasia of HSC, cytopenia with occasional thrombocytosis that resembles MDS/MPN overlap syndrome [123].

#### 2.5.2. ASXL1 and TET2

*ASXL1* plays a role in deubiquitinating histone H2A lysine 119 (H2AK119) through the recruitment of the PRC2 complex. *ASXL1* mutations result in loss of protein function to promote myeloid transformation due to the absence of PRC2 mediated gene repression [124,125]. Approximately 80% of patients with MDS have one or more oncogenic mutations. These mutations include *ASXL1* and *TET2* at mutation frequencies of ~15% and ~22%, respectively, and both are considered as representative mutations found in MDS [62,126,127]. The *ASXL1* gene is localized on chromosome 20q11 and it is one of the frequently mutated genes with prognostic significance [128,129,130]. Some studies have reported *ASXL1* mutation as a gain-of-function mutation that enhances acetylation of H3K122, and this triggers transcriptional activation of *Fos* and *Prdm16* [9,131,132,133]. In contrast, multiple NGS and meta-analysis studies have reported that *TET2* mutations do not possess any prognostic value [134,135]. *TET2* promotes *O*-GlcNAc transferase (OGT) activity by forming the *TET2-OGT* complex to promote enrichment of H3K4me3, contributing to increased transcriptional activity. Therefore, *TET2* mutation causes suppression of OGT activity which directly blocks H3K4me3, resulting in decrease transcriptional activity [134]. Moreover, loss-of-function mutation of *TET2* results in a predominance of 5mC in DNA [134]. The accumulation of 5mC can promote B-cell development and function resulting in activation of innate immune systems, as well as the disruption of DNA methylation homeostasis [136,137]. Despite there being controversies in the functional impact of *TET2* mutations in MDS, it remains as an important epigenetic enzyme [10,134,135].

## 3. Immune Dysregulation

In MDS, a pro-tumor growth microenvironment and the pro-inflammatory signals provided by the immune cells are essential for its pathogenesis. Aberrant signaling pathways like TLR signaling are involved. In the following section, the interactions of adaptive and innate immunity in MDS pathogenesis will be outlined; in particular, immune checkpoint evasions and inflicted inflammation.

### 3.1. Adaptive Immune Dysregulation

The function of immune checkpoints is to regulate antigenic activities and self-tolerance through co-stimulation or direct immune cell inhibition, but tumor cells can evade the tumor surveillance by overexpression of inhibitory proteins. In MDS, immune dysregulation causes autoimmune disease-like features. LR-MDS is characterized by autoimmune-mediated apoptosis, while HR-MDS is characterized by a clonal expansion of selected progenitor lineage [138]. The co-existence of selective cell proliferation and apoptosis of alternative clones complicates the disease pathogenesis. In LR-MDS or early MDS patients, apoptosis signaling is associated with multiple mechanisms: (1) activation of Fas signaling as a result of an increased level of pro-apoptotic cytokines (e.g., TGF-β, IFN-γ and TNF-α); (2) elevation of T-helper (Th) type 17; (3) dysfunctional B-cells; and (4) cytopenic regulatory T-cells (Treg) [139]. Kotsianidis et al. discovered that autoimmunity regulated by Treg is distinct between two MDS risk groups. In comparison with HR-MDS and normal hematopoiesis, CD4^+^ CD25^+^ FOXP3^+^ Treg cells are found dysfunctional and failed to home to bone marrow microenvironment due to downregulation of CXCR4. This leads to decreased self-tolerance hence autoimmunity by T-cells in the bone marrow [140]. This is supported by the additional study from Zou et al. which showed that CD4^+^ Th-cells are deficient rather than CD8^+^ cytotoxic cells. An increased percentage of quiescent memory CD4^+^ and CD8^+^ cells in the peripheral blood of MDS patients is also observed. Hence, a lower age-corrected ratio of CD4^+^:CD8^+^ cells and a significant drop in proliferative index in both T-cell subpopulations have been observed in patients responding to immunosuppressive therapy [141].

While the major effector immune cells in the adaptive immune system lie with CD8^+^ cytotoxic T-cells, major receptors involved in T-cell activation and suppression are grouped into co-stimulatory receptors and co-inhibitory receptors respectively. Co-stimulatory receptors include CD28, 4-1BB, C1D27, ICOS expressed on T-cells, and CD80 and CD86 expressed on antigen presenting cells [142]. On the other hand, co-inhibitory receptors include cytotoxic T-lymphocyte-associated-protein 4 (CTLA4) and programmed cell-death protein (PD1, also known as CD279) are predominantly expressed by T-cells [143,144]. Programmed cell death ligand 1 (PD-L1/CD274) and B7 are ligands of PD-1 and CTLA-4 respectively, which are expressed on antigen-presenting cells (APCs) [143]. The molecular interactions of PD-1/PD-L1 and CTLA-4/B7 axes suppress the immune response. This suppression of T-cell activity serves as an escape mechanism from immune surveillance in several hematological malignancies, including myeloid neoplasms [145,146].

In MDS, studies have shown that PD-1, PD-L1, and CTLA-4 are aberrantly upregulated in MDS patients [146,147]. Yang et al. reported the overexpression of PD-1 in MDS patients. Higher levels of PD-1 have been demonstrated in HR-MDS in comparison with LR-MDS patients [146]. Myeloid cells take advantage of these immune checkpoints by upregulating inhibitory ligands CD47, B7-1, B7-2, and PD-L1 [145,148]. MDS cells have demonstrated the ability to harness some immunosuppressive effects to facilitate their survival and proliferation. For example, T-cells have increased expression of immuno-inhibitory receptors ligands CTLA4, ICOS, PD-1, and T-cell immunoglobulin mucin 3 (TIM-3, also known as CD366) found on MDS patients with treatment-refractory compared with healthy donors [149]. PD-1 and PD-L1 levels are also elevated in patients after treatment of HMAs or upon HMA failure, albeit discovering no significant association between the level of PD-1 expression in response to HMA [146].

In the context of TIM-3 dysregulation which is a distinct AML marker present predominantly on leukemic stem cells (LSCs), TIM3 is absent on normal HSCs [150,151,152]. However, its presence on MDS blasts cells is associated with disease progression and leukemic transformation, and this is evident by the upregulation of pro-proliferative or anti-apoptotic genes [151]. Galectin 9 (Gal-9), one of the ligands of TIM-3 receptor is found excessively expressed on myeloid-derived suppressor cells (MDSCs), exerting an inhibitory effect on immune and inflammatory reactions [153]. The TIM-3/Gal-9 pathway takes part in MDSC-induced T cell exhaustion [150,151,152,153]. It also activates NF-κB and β-catenin signaling and promotes self-renewal in TIM-3^+^ LSCs [154].

### 3.2. Innate Immune Dysregulation

Abnormal innate immunity associated inflammation also contribute to the physio-pathogenesis of MDS. This is evident by overexpression of upregulation of immune related genes in hematopoietic stem or progenitor cells, including TLRs, CD14, and signaling proteins of the NF-κB and MAPK pathways [120,155,156]. As a result, these MDS stem cells increase the production of pro-inflammatory cytokines TNF-α, IL-1, IL-8, and IL-6, contributing to inflammation in MDS. Despite there being a mild discrepancy in dominant TLR responsible for the autoimmune mediated inflammation, TLRs are elevated in CD34^+^ MDS cells. TLR2 is likely responsible for the constitutive apoptosis, and LR-MDS is more prone to this phenomenon in comparison with HR-MDS [117,118,157].

In terms of immune cell, NK cells functions in both innate and adaptive immune response. Unlike early MDS, advanced MDS is characterized by dysfunctional natural killer (NK) cells, immune evasion coupled with increased Treg, therefore, expansion of anti-apoptotic neoplastic cell lineage [139,158,159,160]. The key receptors controlling self-recognition by human NK cells are HLA class I-binding receptors, including the killer immunoglobulin-like receptor (KIR) family as well as the natural killer Group 2A (NKG2A) and leukocyte immunoglobulin-like receptor subfamily B member 1 (LILRB1, also known as LIR-1) [160]. Especially among HR-MDS patients, multiple reports have shown NK cells decreased expression of NKG2D and DNAM-1 [159,161,162]. Carlsten et al. further reported the loss of the potent anti-tumor property of NK cells in vitro and that these NK cells display impaired cytotoxicity towards CD34^+^ MDS blast cells, hence evading tumor surveillance.

For dendritic cells (DCs) and macrophages, which are both the major players in innate immunity, the tumor microenvironment is further complicated in response to the upregulation of proliferative cytokines. While DC is normally responsible for tumor recognition and antigen presentation, cytopenic DC in MDS patients has been reported and is predicted to be defective in activating Treg cells [163]. This finding is supported by recent in vitro studies of DCs isolated from MDS patients (MDS-RA and MDS-RARS) showing significantly lowered levels of both mature and immature DCs coupled with deprived antigen presenting ability to Treg [164]. Moreover, macrophages are found suppressed in MDS and this phenomenon is more profound in HR-MDS than LR-MDS [139]. In general, the macrophage is crucial for phagocytosing, hence clearing cellular debris from aborted differentiation of hematopoietic cells. However, TLR signaling is found to be activated in macrophages via TLR4 overexpression, contributing to inflammasome activation in MDS [155,165]. Han et al. also reported that monocyte-derived macrophages in LR-MDS or intermediate risk MDS patients are found to be reduced despite monocyte counts being higher than in normal individuals. Such suppressed macrophage populations are also inefficient in phagocytosis of abnormal MDS cell clones, producing a pro-tumor growth microenvironment in bone marrow [166].

Most notably, signal regulatory protein alpha (SIRPα), the ligand of CD47, is present on DC and macrophages [167]. With CD47 being a transmembrane protein expressed on the surface of tissue cells and myeloid leukemia cells, CD47 serves as a self-marker for host tissue recognition and evasion of immune response [168,169,170]. During a normal immune response to foreign antigens, expression of the inhibitory ligand CD47 modulates cells into anti-apoptosis and enhances the function of immune suppressor Treg, resulting in antigen-specific T-cell cytotoxicity. When inhibitory ligand expression is low, tumor cell apoptosis can occur and T-cell receptor (TCR) mediates cytolysis, induced by T-cells [171]. With CD47 expression being high in MDS LSCs of HR-MDS compared to those of LR-MDS, binding of CD47 to SIRPα prevents MDS cells from phagocytosis and hence promotes selected clonal expansion, contributing to a HR-MDS phenotype [168,169,170].

## 4. Treatment of MDS

### 4.1. Hypomethylating Agents (HMA)

The first generation of HMA was developed as conventional cytostatic therapy back in the 1960s [172]. The use of HMA drugs in MDS aims to restore the expression of tumor suppressive genes silenced by promotor hypermethylation [173,174]. The current generation of HMA drugs are AZA (5-azacytidine) and DEC (5-aza-2′-deoxycytidine). AZA is administered subcutaneously at a dose of 75 mg/m^2^ for 7 days every 28 days, and DEC is given intravenously at a dosage of 15 mg/m^2^ every 8 h for 3 days with repetition every 6 weeks. Both drugs have shown beneficial effects in MDS and have been approved by the US Food and Drug Administration (FDA) since 2004 and 2006, respectively [175,176]. A 5-day regimen of DEC at 20 mg/m^2^ at a 28-days interval that allows easier administration was later approved by the FDA in 2010 as the clinical standard [173,174]. DEC has been reported to stimulate NK cell responsiveness to IL-2 stimulus while AZA can impair NK cell activities through IFN-γ modulation [177]. Allogeneic immune reactions of donor lymphocyte infusions by DC have also been reported to increase through HMA treatment [178]. In contrast, the bone marrow microenvironment, such as mesenchymal stromal cells (MSC), was found suppressed upon HMA treatment in MDS patients [179].

There are studies that have demonstrated the immunomodulatory effects of HMA in MDS. Gomez et al. evaluated the hematopoietic architectures of MDS cells before and after HMA treatments and discovered distinct sub-populations, being CD34^+^ and CD38^+^, upon disease progression [180]. CD38 expression on CD8^+^ T-cells was found to have a negative correlation with IFN-γ and abundance of CD8^+^ T-cells after the introduction of HMA indicated decreased T-cell activity [181]. Another study demonstrated that a decrease in CD8^+^ cells is associated with the introduction of DEC with anti-PD1 treatment [182]. Early research has also shown the induction of Tregs via demethylation of FOXP_3_ promoter by HMA, this led to investigation of how HMA affects the functions of NK cells [183,184].

**Figure 5:**
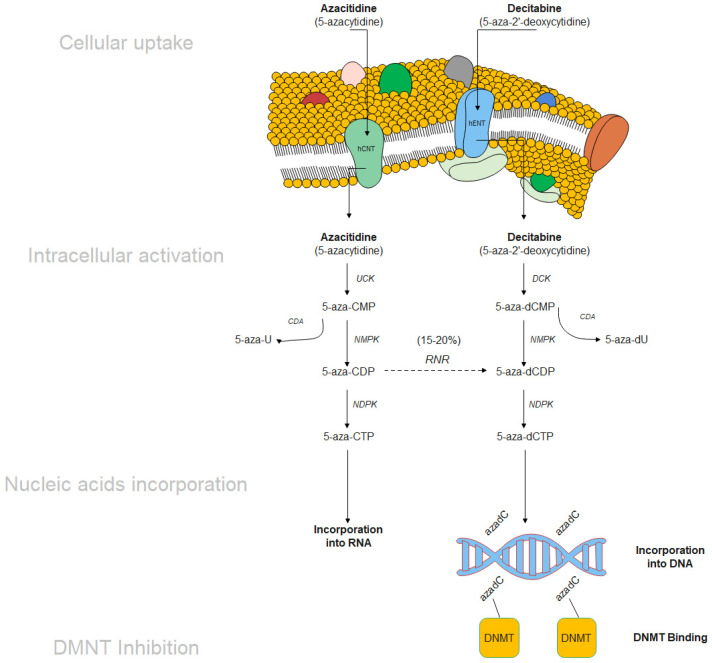
Schematic representation of AZA and DEC uptake and metabolism. UCK—uridine-cytidine kinase, DCK—deoxycytidine kinase, CDA—cytidine deaminase, NMPK—nucleoside monophosphate kinase, NDPK—nucleoside diphosphatase kinase, RNR—ribonucleotide reductase.

The molecular mechanism of HMA comprises of cellular uptake, intracellular activation, nucleic acids incorporation, and DNMT inhibition resulting in DNA hypomethylation (Figure 5). The cellular uptake is regulated by two different transporters: (1) human concentrative nucleoside transporter (hCNT) for AZA intake, and (2) human equilibrative transporter (hENT) for DEC intake [185]. Enzymes catalyzing the rate limiting step are 1-uridine-cytidine kinase (UCK) and deoxycytidine kinase (DCK), which produce 5-azacitidine-triphosphate (5-aza-CTP) from AZA and 5-aza-2′-deoxycytidine-triphosphate (5-aza-dCTP) from DEC, respectively. HMA is considered as an S-phase specific drugs due to its nature of incorporation into DNA during cell replication.

Upon intracellular activation, DEC exclusively incorporates into DNA only and AZA primarily incorporates into RNA. Meanwhile, 15–20% of AZA intake can be converted from 5-aza-CDP to 5-aza-dCDP through ribonecleotide reductase (RNR), which then can be integrated into the DNA. After a series of phosphorylation events give rise to the 5-aza-CTP and 5-aza-dCTP for AZA and DEC, respectively, which leads to incorporation of RNA and DNA resulting in irreversibly bind to maintenance DNMT1 resulting in degradation of DNMT1 and DNA de-methylation [179]. DNA demethylation can lead to the reactivation of abnormal silenced genes, involving multiple pathways, such as angiogenesis, apoptosis, differentiation, and DNA repair [179].

### 4.2. Mechanisms of Resistance to Hypomethylating Agents

In spite of the good initial treatment responses to HMA, 40% of MDS patients will develop resistance to HMA [179,186]. There are two types of HMA resistance: primary resistance, which refers to a patient with no improvement after 4–6 cycles of treatment, and secondary resistance that refers to disease relapses after long-term treatment. Various studies have been performed to study primary and secondary HMA resistance. Qin et al. demonstrated that resistance to DEC in most cancer cell lines has downregulated genes involving in the uptake and activation of DEC, while they also noticed a high expression of cytidine deaminase (*CDA*) [186,187]. The loss of *DCK* and mutations of *UCK2* can cause resistance to DEC and AZA, respectively. Unfortunately, pre-clinical evidence of HMA resistance in MDS is relatively unclear. For DEC resistance, MDS patients with primary resistance present a high CDA to DCK ratio resulting in the inactivation and decrease of 5-aza-dCTP. In MDS patients with secondary resistance, upregulation of mutated *DCK* mRNA with suppressed activities of DEC. For AZA, a downregulation of *UCK1* in MDS AZA resistant patients is reported but not *UCK2*, postulating the two enzymes do not have identical relevance to AZA resistance [188,189,190].

In terms of immunomodulation during HMA resistance, there are studies that have demonstrated failed immune response being correlated with high expression of *PD-1*, *PD-L1*, *PD-L2* and *CTLA-4* in MDS. Treatment with DEC results in a dosage-dependent upregulation for these genes, and partial demethylation of PD-1 [146]. Ørskov et al. also demonstrated that demethylation of PD-1 promoter correlates with significantly inferior response rates during HMA treatment in MDS. They also reported that HMAs induce PD-1 expression on T cells in the MDS microenvironment, thereby impeding the immune response against the MDS blasts [191].

### 4.3. Next Generation HMA—Strategies to Overcome HMA Resistance

Although AZA and DEC currently remain standards of care in MDS, their short half-life and poor oral availability prompted persistent efforts for the development of novel HMAs and combinations [187]. CC-486, an oral formulation of AZA, was developed to enhance the ease of administration and dose adjustment [187]. In a study, MDS patients presented a reduction in DNA methylation induced by the current 7-day AZA regimens that was subsequently reversed towards the end of the cycle [192]. In contrast, persistent reduction of methylation was achieved by extended dosing of CC-486 for either 14 or 21 days [193]. The use of the 14- and 21-day regimens in patients with LR-MDS produced an encouraging overall response rate (ORR) of 38% alongside a tolerable safety profile. The most frequent grade 3–4 adverse events were neutropenia, anemia, and gastrointestinal disturbances [194]. In a placebo-controlled randomized phase III trial, CC-486 significantly enhanced red cells and platelet improvement rates at the cost of increased incidence of adverse events compared to placebo (90% vs. 73%) [195]. In spite of the higher infection-related mortality rate in the CC-486 arm in the first 56 days, the overall mortality rates remained similar between the two arms [196]. Alternative treatment may overcome adaptive responses due to repetitive use of HMA. Recent clinical trials have also explored novel HMAs along with combination therapy as potential strategies to overcome HMA resistance [195].

#### 4.3.1. Guadecitabine (SGI-110)

Guadecitabine (SGI-110) is a dinucleotide of DEC and deoxyguanosine that is administered via subcutaneous injections [187]. A DEC analog is resistant to deamination by CDA, and is hence more effective with easier administration given the less-frequent dosing requirements compared to AZA and DEC [197]. The prolonged duration of action of this agent can be accounted for by its resistance towards CDA mediated degradation [187]. A multicenter phase I study among relapse/refractory (R/R) AML and MDS patients highlighted its clinical efficacy, where 22% (2 out of 9) of MDS patients showed marrow complete responses [198]. A phase II study of guadecitabine in HR-MDS patients with AZA resistance demonstrated an ORR of 14.3% with significant improvement in survival among responders [199]. However, in the randomized phase III ASTRAL-3 trial, guadecitabine failed to improve the survival of previously treated MDS patients when compared to standard treatments [200]. This agent was generally well tolerated, with the most common grade 3 or above adverse events being febrile neutropenia, myelosuppression, and infections [198,199].

#### 4.3.2. ASTX727

ASTX727 is an orally available combination of DEC and CDA inhibitor cedazuridine. Its impressive activity is first elucidated in a multicenter phase I/II study among MDS and CMML patients, where non-inferiority of ASTX727 towards IV DEC is established, along with a CR rate of 18% and transfusion independence rate of 49% [201,202]. In the randomized phase III ASCERTAIN study among MDS patients, comparable demethylation activity and safety profiles between ASTX727 and IV DEC were demonstrated [195]. These satisfactory results have prompted FDA-approval of ASTX727 for previously treated and untreated, de novo and secondary MDS with specific FAB subtypes (RA, MDS-RARS, MDS-RAEB, and CMML) and IPSS scores (intermediate-1, intermediate-2, and high-risk) in 2020. ASTX727 has a favorable safety profile, with febrile neutropenia and infections being the most common serious adverse events [201,202,203].

### 4.4. Targeted Therapy in Combination with Hypomethylating Agents

Although HMA treatments have survival benefits and are the current standard of care, many MDS patients will not garner a response from therapy. For those who do respond, most responses are not durable, and the only hope for treatment is allo-HSCT. New therapies to combat HMA resistances are urgently needed. Several of these small molecules have demonstrated the ability to augment the response rates of HMA, including complete remission (CR) rates, in both the front line and refractory settings. Clinical trials of targeted therapy for MDS patients are mainly based on the safety and efficacy data demonstrated in AML patients. This section focuses on discussing the rationale and application of co-administration of targeted therapy and HMAs in MDS patients.

#### 4.4.1. BCL-2 Inhibitors

Venetoclax, approved by the FDA, is a BCL-2 inhibitor that can be used in combination with HMAs in HR-MDS patients and has been reported to have a good therapeutic response in a case study as a monotherapy (ClinicalTrials.gov (accessed on 21 September 2021) Identifier: NCT02966782) [204]. It acts as a BH3 mimetic that impedes the binding of BH3 proteins to BCL-2, hence releasing pro-apoptotic BAK and BAX proteins [205,206]. This results in mitochondrial outer membrane permeabilization (MOMP) with the release of cytochrome C into the cytoplasm, leading to the formation of cytosolic apoptosome complex, caspase activation, and subsequent cellular apoptosis [207].

More importantly, dose optimization co-treatment of venetoclax and AZA could effectively spare hematopoiesis without affecting its ability to target malignant cells [208]. Encouraging results have been observed in clinical trials. A phase 1b trial on treatment-naïve HR-MDS patients demonstrated an ORR of 74% and progression-free survival (PFS) of 59% [209,210]. This finding was further validated in another study that showed a compatible ORR with a majority of patients being bridged to allo-HSCT [211]. The phase 3 VERONA trial is currently underway to further assess the safety and efficacy of combination therapy (ClinicalTrials.gov Identifier: NCT04401748) [212]; however, it should be noted that cytotoxic effects might be aggravated by concomitant CYP3A4 inhibitors [206,208]. For instance, dose-adjustment of venetoclax is required with concomitant use of triazole, a frequently used CYP3A4-inhibiting anti-fungal in MDS patients [206]. In addition, granulopoiesis is particularly suppressed in combination therapy, febrile neutropenia is, therefore, a commonly exhibited adverse effect [208,209,211].

Pollyea et al. reported that the use of ventoclax with AZA disrupts energy metabolism in LSC, resulting in more durable remissions by inhibiting amino acid metabolism, leading to cell death. The effects of combinational therapy yielded much better results in comparison with conventional treatments [213]. Two years later, the second part of this study demonstrated resistance forming from a combinational therapy of venetoclax/AZA, which failed to eradicate LSCs in R/R patients caused by elevated nicotinamide metabolism. Metabolomic analysis reveals elevated nicotinamide causes in the activation of both amino acid metabolism and fatty acid oxidation resulting in oxidative phosphorylation. This provides means for LSCs to evade the cytotoxic effects of venetoclax/AZA therapy [214].

#### 4.4.2. IDH1/2 Inhibitors

Ivosidenib is a potent orally available *IDH1* inhibitor. It suppresses mutant *IDH1* and hampers the synthesis of oncometabolite 2-HG, resulting in aberrant DNA and histone hypermethylation, differentiation arrest of the myeloid lineage, and initiation of leukemogenesis [215,216,217]. A phase 1 dose escalation and expansion trial was conducted on *IDH1*-mutated R/R MDS patients [215,218,219]. An ORR of 91.7% was entailed and 60% of patients remained in CR at 12 months, revealing the favorable response of ivosidenib in R/R MDS patients [215,218,219]. Olatusidenib (FT-2102) is another potent *IDH1* inhibitor that restores cellular differentiation in MDS patients [210,220]. Clinical responses were seen in 33% and 73% with olatusidenib monotherapy and combination therapy, respectively, in a phase 1/2 trial [220]. Safety and tolerability were depicted [220,221]. More clinical trials are currently underway (ClinicalTrials.gov Identifier: NCT02719574).

Enasidenib specifically targets *IDH2* and could be used in MDS patients harboring *IDH2* mutations. The effectiveness of enasidenib was recognized in the AG221-C-001 trial, where it not only gained FDA approval in 2017, but also evoked further investigations in *IDH2*-mutated R/R MDS patients [222,223,224]. OS was extended by around 3 times from 5 months to 16.9 months [222]. A phase 2 clinical trial was performed to further evaluate the tolerability and efficacy of enasidenib as a monotherapy or as a combination therapy with AZA [225,226]. Hopeful results as manifested with 100% HMA-naïve HR-MDS patients responding to concurrent AZA-enasidenib therapy. Fifty percent of patients who were previously HMA-resistant were responsive to enasidenib monotherapy. In addition, most adverse effects were manageable, implying enasidenib is a tolerable novel agent in HR-MDS patients [225,226]. Further clinical results are anticipated (ClinicalTrials.gov Identifier: NCT03744390; NCT03383575; NCT03839771).

#### 4.4.3. FLT3 Inhibitors

Midostaurin is a first-generation type 1 *FLT3* inhibitor that targets both *FLT3-ITD* and *FLT3-TKD* mutations. Concomitant use of midostaurin and AZA only showed an ORR of 26% in a phase 1/2 study in *FLT3*-positive HR-MDS and AML patients [227]. As *FLT3* mutation is associated with leukemogenesis, it is suggested that blast reduction is the most significant observation. Clinical response remains limited and was neither profound nor sustained enough to achieve CR [228]. Gilteritinib, a second-generation type 1 *FLT3* inhibitor, was also investigated in MDS-EB-2 patients although the results have yet to be published (ClinicalTrials.gov Identifier: NCT04027309). Its use with venetoclax and AZA is also warranted (ClinicalTrials.gov Identifier: NCT04140487).

In contrast to midostaurin and gilteritinib, sorafenib is a first-generation type 2 *FLT3* inhibitor with explicit *FLT3-ITD* inhibition. To date, the clinical efficacy of sorafenib has been limited and the results have been disappointing [118,229,230]. Co-treatment of sorafenib and a low dose cytarabine only demonstrated 10% ORR. The discouraging results might be explained by the use of low-dose cytarabine instead of HMAs, which is efficacious in MDS patients [118,230]. In addition, the off-targeting effect of sorafenib on vascular endothelial growth factor receptor (VEGFR) and platelet-derived growth factor receptor (PDGFR) give rise to unwanted adverse effects, such as hand–foot skin reaction [118]. Quizartinib is a second-generation type 2 *FLT3* inhibitor. Multiple clinical trials with HMAs and/or FLT3 inhibitors are underway (ClinicalTrials.gov Identifier: NCT01892371; NCT03661307; NCT04493138). An interim report of the phase 1/2 trial showed that quizartinib exhibits an ORR of 67% in *FLT3-ITD*-mutated patients, which is higher than that of monotherapy [231]. Hence, this provides hope for *FLT3*-mutated MDS patients, especially those who are not candidates for allo-HSCT.

### 4.5. Splicing Inhibition and TP53 Modulation

The SF3b subcomplex is a component commonly targeted by splicing inhibitors [232,233]. They prevent the binding of SF3b subcomplex to pre-mRNA, leading to the blockade of spliceosome assembly [232,233]. Among the numerous splicing inhibitors, E1707 and H3B-8800 are the only two spliceosome inhibitors being tested in clinical settings [234]. E1707 is a pladienolide derivative targeting SF3B1 and was first evaluated in a phase 1 trial for advanced solid tumors [235,236]. However, further clinical investigation is discouraged as a patient developed optic neuritis. Nevertheless, its association with splicing inhibitors has not yet been corroborated [235]. H3B-8800 is a recently developed orally available splicing modulator that also binds to SF3b complexes, altering mRNA splicing and hence lethality [232,233,234,237]. A phase 1 clinical trial has been conducted, showing safety and a predictable pharmacokinetic profile in MDS, CMML, and AML patients [238]. Decreased transfusion independence has been also observed in 14% of patients [238]. Due to the investigation into the use of splicing inhibitors being early, the exciting results prompt further exploration. Inhibition of spliceosome mutations might be a potential therapeutic option in MDS patients in the future.

Eprenetapopt (APR-246) is a novel small molecule that spares normal cells, while it covalently binds to mutant and wild-type p53 to thermodynamically stabilize p53 mutants for reactivation of its functions and restoring the conformation of misfolded p53 wild-type proteins to ultimately eradicate leukemic cells [239,240,241,242]. In a phase Ib/II study, combination of eprenetapopt with azacitidine resulted in an ORR of 73% in MDS with 50% achieving CR and 58% achieving a cytogenetic response [243].

### 4.6. Immune Checkpoint Inhibition in Combination with HMA

#### 4.6.1. Anti-PD-1, Anti-PD-L1, Anti-CTLA-4

The elevated levels of PD-1 and PD-L1 have been found in patients after treatment of HMAs or in the case of HMA failure, albeit discovering no significant association between level of PD-1 expression with response to HMA [146]. This sheds light on immune checkpoint molecules as therapeutic targets for the development of immune checkpoint inhibitors and as potential agents in refractory disease. Pembrolizumab (MK-3475) and nivolumab are both humanized monoclonal antibodies (mAbs) that serve as anti-PD-1 inhibitors. Durvalumab (MEDI4736) and atezolizumab, on the other hand, act as anti-PD-L1 inhibitors. Ipilimumab is an anti-CTLA-4 inhibitor (Table 1).

Monotherapy with pembrolizumab or ipilimumab exhibited suboptimal responses in HMA-resistant patients with ORRs of 4% and 3.4%, respectively (NCT01953692) [244,245]. Nevertheless, a phase 2 trial done by Chien et al. showed the potential anti-leukemic effect of pembrolizumab with AZA in HMA-resistant patients (NCT03094637) [246]. Another phase 2 trial conducted by Garcia-Manero et al. also preliminarily observed superior therapeutic effects for nivolumab or ipilimumab as a combination therapy with AZA, with ORRs of 75% and 71%, and median survivals of 12 months and not reached, respectively. A better synergistic response was observed in ipilimumab than with nivolumab with AZA [247]. However, they both performed poorly as single agents (both with median survivals of 8 months), which was supported by another phase 2 study showing ORRs of 0% and 22% in nivolumab and ipilimumab, respectively [247,248]. The use of nivolumab with AZA also displays anti-leukemic effect in de novo MDS patients with an ORR of 69% [248]. Notably, the addition of the anti-PD-L1, durvalumab, to AZA also augmented the ORR in HR-MDS, as demonstrated in a study performed by Zeidan et al. (ORRs: 61.9% vs. 47.6%) (NCT02775903) [249]. Atezolizumab, on the other hand, has demonstrated limited efficacy with unfavorable safety when engaged with AZA [250]. Clinical trials on the combination of pembrolizumab with entinostat, a HDAC inhibitor, or DEC are underway (NCT02936752, NCT03969446). Other trials combining nivolumab, ipilimuma or durvalumab with HMA are also being evaluated (NCT02117219, NCT02281084, NCT02530463, NCT02890329, NCT03092674).

#### 4.6.2. Anti-TIM-3

The introduction of anti-TIM-3 antibodies put an end to the proliferation of leukemic blasts. This finding eventually led to the development of mAb antagonizing TIM-3 receptors as a possible novel treatment for HR-MDS [251]. A phase 1b study carried out by Borate et al. demonstrated that the use of DEC with sabatolimab in HR-MDS can achieve 50% CR and molecular CR (mCR) [252]. In addition, another phase 1b trial conducted by Brunner et al. showed an ORR of 62.9% with superior response in very high risk IPSS-R than high risk IPSS-R patients (84.6% vs. 50%) [251]. A phase 1 trial is currently in progress, studying the safety and tolerability of sabatolimab alone and in combination with AZA or DEC (NCT03066648). Phase 2, STIMULUS-MDS1 (NCT03946670), and phase 3, STIMULUS-MDS2 (NCT04266301), trials are also underway, evaluating the efficacy of the combination of sabatolimab with HMAs [153]. This prompts further development of several impending phases 2 trials, STIMULUS-MDS3 and STIMULUS MDS-US, evaluating the efficacy of sabatolimab in conjunction with HMAs in HR-MDS (NCT04812548, NCT04878432).

#### 4.6.3. Anti-CD47

With CD47 overexpressed in MDS and the binding of CD47 to SIRPα prevents MDS cells from phagocytosis, the blockade of such interactions promotes antibody-dependent cytotoxic phagocytosis of the tumor cells opsonized with antibodies [253]. This has led to the introduction of magrolimab, TTI-621, and CC-90002 as anti-47 mAbs for therapeutic advancement in MDS [168,169,170]. Magrolimab (Hu5F9-G4) is a mAb targeting malignant cells via macrophage phagocytosis. It serves as a checkpoint inhibitor of macrophages that exhibits anti-phagocytic properties [253]. Despite the limited efficacy of magrolimab as a monotherapy, its use as a combinatory drug exhibits potential synergistic effects. Sallman et al. conducted a phase 1b trial on the use of magrolimab with AZA and evaluated its efficacy in MDS and AML patients. Objective response was seen in all de novo MDS patients in the study. The combination significantly shortened the time to response by a median of 1.9 months when compared with AZA alone [254]. This led to the expansion of the trial, which has demonstrated a pronounced objective response of 91% in HR-MDS patients, suggesting that magroliumab is a promising therapeutic candidate for the development of future novel drugs (NCT03248479) [255]. TTI-621, also known as signal regulatory protein α-Fc (SIRPαFc), also entered a phase 1 trial, evaluating its effectiveness as a single agent in MDS (NCT02663518). Currently, a phase 3 trial, ENHANCE, is underway, comparing the efficacy of the synergy between magrolimab and AZA and AZA alone (NCT04313881) [196]. Other ongoing trials are also under investigation (NCT03248479). AK117, a novel IgG4 mAb antagonizing CD47, was also tested in a phase 1/2 trial for safety and efficacy assessment in HR-MDS (NCT04900350).

### 4.7. Adoptive T-Cell Therapy with HMA or as Monotherapy

Adoptive T-cell therapy is the infusion of T-lymphocytes into a patient’s body to directly target an antigen, resulting in an increase of cytotoxicity for the target cell. T-cell transfers can be autologous or allogeneic, which can be an unmanipulated transfer (donor lymphocyte infusion) or a manipulated transfer, such as ex-vivo priming with onco-antigens, chimeric antigen receptor (CAR) T-cell constructs and modified T-cell receptors. For example, by immunizing with antigens, there are in vitro studies conducted to find ways to manipulate lymphocytes and target myeloid antigens for patients with MDS. There are only two phase 1 clinical trials of CAR T-cell therapy on intermediate to HR-MDS patients or patients failing HMA (ClinicalTrials.gov Identifier: NCT-03258359; NCT02203825); results have not yet been published.

#### 4.7.1. Anti-CD123

Steven et al. demonstrated that cell surface antigen CD123 is overexpressed on MDS stem cells [256], with a gradual increase of CD123 expression from LR-MDS, intermediate to HR-MDS [257]. CD123 CAR-T cells eradicated CD123^+^ MDS stem cells in vitro as a proof-of-concept for a valid treatment for high risk MDS patients. While eradicating CD123^+^ MDS stem cells may substantially reduce disease burden residuals of CD123^negative/low^ the MDS stem cells that were left behind are still of concern. Li et al. demonstrated that CD34^+^CD123^+^ stem population is not as abundant in LR-MDS and intermediate risk MDS in comparison with HR-MDS patients, postulating that MDS stem cells in these patients will not be effectively targeted by CD123 CAR T-cells [257].

#### 4.7.2. Anti-NKG2D

The natural killer group 2 receptors (NKG2D) are positive immunomodulatory proteins found on NK and CD8^+^ T cells. When there is a presence of intracellular stress, such as DNA damage, infections, inflammation, and toxins, cancer cells express high levels of the MHC I chain-related protein A/B (MICA/B), which are ligands for NK2GD hence inducing tumor elimination by NK-mediated or CD8^+^ T-cell [258,259]. In ~30% of MDS patients, protein expression of both MICA and MICB is found in CD34^+^ cells. At the same time, NKG2D is downregulated in MDS patients and is correlated with impaired NK-mediated cytotoxicity. Moreover, impaired NK function in MDS has been reported with significant associations to higher IPSS risk, abnormal karyotype, excess blasts percentage, and marrow hypercellularity [260]. Other pre-clinical models have also been reported on the extended potential of NKG2D CAR T-cells, such as in the overexpression of the receptor to overcome natural inhibition mediated by MICA [261,262,263] and the co-stimulation of CD28 for T cell activation and survival through the activation of DNAX-activating protein of 10 kDa (DAP10) [264,265]. These data collectively confirm the evasion of NK surveillance by MDS cells, prompting the development of CAR T-cell therapy targeting this axis [266].

Genetically engineered T-cells to express NKG2D have demonstrated the potential to specifically target, not just cancer cells, but also Tregs and myeloid-derived suppressor cells within the tumor microenvironment [267]. Clinical trials of NKG2D CAR T-cells in MDS patients showed the overexpression of the ligands for NKG2D [267,268]. Ongoing phase I trials (ClinicalTrials.gov Identifier: NCT02203825) are now underway but preliminary reports have demonstrated NKG2D CAR T-cells with transient hematologic improvement against autologous tumor cells in vitro of MDS patients [265].

### 4.8. Donor-Derived Lymphocytes against Tumor-Associated Antigens

Relapse following allogeneic HSCT is associated with a dismal outcome [269]. Unmanipulated donor cell infusion (DLI) or second allogeneic HSCT may be considered but responses are often unpredictable [270,271]. Patient-specific DLI with tumor-associated antigen (TAA) stimulation ex vivo selects for an enriched, polyclonal CD4^+^ and CD8^+^ specifically target myeloid malignancies have shown promises in aiding advanced treatment in MDS. Lulla et al. were able to mitigate GVHD with non-manipulated DLI by selecting T-cells with specific MDS antigens that are not typically present on normal host cells, such as NYESO1, PRAME, Survivin, and WT1. Twelve patients who received TAA-cell prophylaxis (n = 12) had 4 relapses within 1 year of infusion, of which the 11 patients that received tailored DLI TAA-T cells remain alive. Respondents to the treatment had a measurable expansion of leukemia antigen specific T-cells which persisted for over 9 months with no experience of any infusion related GVHD [272].

TAA-DLI has shown potential in disease control compared with unselected donor T cell infusion in post-transplant settings; however, its limitations lie with the productivity of the T-cells as its highly dependent on donor availability. The utilization of autologous CAR T therapy in MDS remains uncertain. There are many problems that must be overcome such as the inability to persist with T cell response memories problematic for this to be a make a valid therapy option for HR-MDS. Through CAR T cell expansion processes, problems, such as the immunosuppressive and impair immune effects, can be rectified and reinforce the central dogma of the bone marrow microenvironment in MDS [273,274]. NK cell therapy has also been reported to be a well-tolerated therapy for HR-MDS or patients refractory to chemotherapy [158]. Although tested in only a very few MDS patients, Björklund et al. reported that infusion of haploid identical NK cells from a healthy donors can reduce the allelic burden of tumor cell clones with high risk *ASXL1* and *RUNX1* mutations. MDS patients responding to this treatment also shows undetectable mutations upon therapy.

## 5. Conclusions

MDS is a clonal hematological disease with substantial genetic and epigenetic complexity and heterogeneity. The genomic profile should be incorporated into the personalized prognostic assessment of MDS and therapeutic targeting with novel agents. To overcome HMA resistance, combinatorial approaches involving novel agents and HMA are required. These include molecular targeted therapy and immune checkpoint inhibition.

## Figures and Tables

**Table 1:** List of clinical trials on novel agents and combinatorial treatment for MDS.

Drug		Phase	Disease Subtype	Regimen	Status	Clinical Trial Identifier
Immune Checkpoint Inhibitors						
Pembrolizumab	Anti-PD1	1	Hematologic malignancies	Pembrolizumab	Completed	NCT01953692
		1	MDS	Pembrolizumab + Entinostat	Active, not recruiting	NCT02936752
		2	MDS	Pembrolizumab + AZA	Recruiting	NCT03094637
		1	ND/RR AML/MDS	Pembrolizumab + DEC	Recruiting	NCT03969446
Nivolumab		2	MDS, R/R MDS	Nivolumab + AZA, Ipilimumab + AZA	Recruiting	NCT02530463
		2/3	AML, MDS	AZA + Nivolumab/Midostaurin, DEC + Cytarabine	Active, not recruiting	NCT03092674
Durvalumab (MEDI4736)	Anti-PD-L1	1	MDS	Durvalumab, Durvalumab + Tremelimumab	Completed	NCT02117219
		2	MDS	Durvalumab + AZA	Active, not recruiting	NCT02281084
		2	AML, MDS	AZA, AZA + Durvalumab	Active, not recruiting	NCT02775903
Ipilimumab	Anti-CTLA-4	1	R/R MDS, AML	Ipilimumab + DEC	Recruiting	NCT02890329
Sabatolimab (MBG453)	Anti-TIM-3	1	AML, HR-MDS	MBG453, or in combination with PDR001/DEC/AZA	Active, not recruiting	NCT03066648
		2	HR-MDS	MBG453 + HMA	Active, not recruiting	NCT03946670
		3	HR-MDS, CMML-2	MBG453 + AZA	Recruiting	NCT04266301
		2	HR-MDS	Sabatolimab + AZA + Venetoclax	Not yet recruiting	NCT04812548
		2	HR-MDS	Sabatolimab + AZA/DEC	Not yet recruiting	NCT04878432
TTI-621 (SIRPαFc)	Anti-CD47	1	Hematologic malignancies, solid tumors	TTI-621 for MDS	Recruiting	NCT02663518
Magrolimab		1	Hematologic malignancies	Magrolimab, Magrolimab + AZA	Recruiting	NCT03248479
		3	MDS	AZA, AZA + Magrolimab	Recruiting	NCT04313881
AK117		1/2	MDS	AK117 + AZA	Recruiting	NCT04900350

Abbreviations: ND, newly diagnosed.
